# Suppurative Disease of the Maxillary Sinus

**Published:** 1902-08-15

**Authors:** Herbert M. King


### SUPPURATIVE DISEASE OF THE MAXILLARY SINUS.

BY HERBERT M. KING, M.D.

Read before the Michigan Dental Association, June 10, 1902.

Suppurative disease affecting the accessory sinuses of the nose is a much more common condition than is generally recognized, notwithstanding the abundance of literature on the subject.

For the present purpose we are to consider this condition as affecting the antrum alone, and particularly as it relates to dentistry and oral surgery. My few remarks, therefore, will be chiefly along this line and, without entering into an exhaustive study of the pathologic features are intended merely for the purpose of bringing out a practical discussion of the subject in its clinical aspects.

Empyema of the antrum may be acute or chronic. It not infrequently happens that the acute condition fails to subside entirely and leaves a chronic suppuration, most obstinate in its response to treatment, or a chronic suppuration may come on gradually from the start and never appear as an acute condition.

The first class of cases owe their peculiar behavior to the character of the infection, the constitutional dyscrasia or the anatomical anomolies of the cavity itself, which, as you know, may be so divided into separate illy communicating cells by thin boney septa as to make perfect drainage very difficult if not impossible.

In the second class, an infection extending insidiously from a diseased nasal mucosa by continuity of structure into the antrum, may occur without occlusion of the ostium maxillare so that acute symptoms of inflammation naturally never appear; or a diseased bicuspid or first molar whose root pierces the floor of the antrum may abscess and owing to the readiness with which the soft tissues covering it yield to pressure pus forming at such a point gradually fills the antrum and overflows into the nasal chamber without acute symptoms. It may, indeed, be set down in this wise, acute local symptoms appear only when there is interference with drainage and consequent pressure from accumulating pus. Hence it is that not infrequently empyema, in an anatomically normal antrum, arising from one or more diseased teeth, may be, and I am sorry to say, very often, is treated for a nasal catarrh, until for one reason or another the nasal opening of the sinus becomes occluded and local and constitutional symptoms of sepsis supervene.

There is, however, a form of suppurative disease of the antrum, secondary to acute infections or other pathologic conditions of the nasal chambers proper, which can neither, strictly speaking, be termed acute nor chronic. For the sake of convenience I classify this form as "subacute." It often supervenes upon an influenza, or other acute infection of the nasal mucosa. It is not rarely a complication of nasal polypus, and I have known it to follow traumatism. It yields readily to treatment, or subsides without treatment at all in from one to four weeks, and is I think the most frequent of antrum empyemas; but for obvious reasons it need not be considered in the present discussion.

In the other forms I have always contended that abnormalities in the teeth or their surrounding structures must be considered the most common etiological factor. I can see no reason now to alter the views expressed in a paper presented at the Michigan State Medical Society Meeting in 1894 from which I quote * * "My own experience, together with what I have been able to glean from reports extant upon the subject, convinces me that suppurative disease of the antrum is of very much more frequent occurrence than that of all the other accessory cavities put together, but I can not well see why the antrum should be more often affected from causes arising within the nose than should the other sinuses, subjected as they are to identical influences so far as the nose is concerned; and it is the consideration of these facts, supported by my experience in these complications, which strongly inclines me to the belief that to diseases of the teeth and surrounding structures we must look for the most frequent causes of empyema of the antrum, while to intranasal disease in the majority of instances may be attributed a like condition occurring in the other accessory cavities." The dentist, therefore, will frequently have the earliest opportunity of making a diagnosis of antrum disease, and when a patient consults him complaining of neuralgic pain in the face and eye (with or without local swelling) and tenderness upon pressure against the upper teeth from first biscuspid to second molar he should always have in mind the possibility of this condition. He should inquire as to the presence of a purulent discharge from one naris which often the patient ascribes to a "cold," and he should examine carefully as to whether any of the teeth, whose roots come into proximity with the antrum, are diseased, or have their pulps devitalized. It must be borne in mind that in this disease as in most others, success in treatment largely depends upon early diagnosis. In this matter of diagnosis, so far as the antrum is concerned, there are few real difficulties to contend with. Permit me to again quote from my paper of 1894, * * * "The Voltolini method of transillumination by means of a small incandescent lamp promised much toward making diagnosis in these cases easy, but I must confess that I have not found it a very reliable test, except in demonstrating the presence of pus in the frontal sinuses. Where the ethmoidal cells and maxillary sinuses are suspected it has been of little value in my hands, and, to be brief, a chronic purulent discharge from the nares, more or less profuse, especially if it be unilateral and we can eliminate syphilis, foreign body, neoplasm, and simple purulent rhinitis, rarely if ever seen in adult life, is a sufficiently suspicious sign to warrant us in taking the only step which will make the diagnosis absolutely correct—namely, abstracting pus from the suspected cavity. This may be effected by means of small aspirators constructed of proper shape and strength in the case of any of the cavities in question except the frontal sinuses, without excessive pain and with little or no danger of untoward results if the suspected cavity should prove healthy. It is hardly necessary to say that where suspicion falls upon the antrum and a carious tooth exists upon the affected side, removal of the diseased tooth, especially if it should chance to be a first molar or second bicuspid, is the indication, when, if this alone does not effect an entrance, it is a very easy matter to do so through the alveolus; should the teeth be perfectly sound however I should aspirate through the thin lateral wall of the nose with a strong curved trocar." Where, however, the antrum affected is not a single cavity, but as above mentioned, divided by bony septa into two or more cavities communicating if at all but poorly with each other and with the naris the case may present more difficulty of diagnosis. I have seen several such and confess to no little confusion before a correct diagnosis was reached. A case of this nature coming under my observation a few years ago it may not be amiss to relate briefly.

The patient, a man between twenty and thirty years of age, had been in rather delicate health for several years. One year prior to my examination he consulted his dentist because of a hard swelling above the gums on the left side of the face which he correctly supposed was due to a diseased first molar. The dentist ordered a poultice for a day or two when the mass softened and upon opening it a considerable quantity of foetid pus was evacuated, the abscess was then treated through the alveolar opening with indifferent success, and in March 1897 his dentist brought the patient to me for consultation. Upon examination it was found impossible to pass a probe into the antrum through the fistula. And there was only a scant and intermittent discharge of pus into the left naris but from the history of the case and the general appearance at the time of examination I agreed with the dentist that it was a case of empyema of the left maxillary sinus arising from disease at the root of the first molar on the affected side. This tooth was extracted and with little difficulty an opening was effected into the antrum through the pocket in the alveolus of the anterior root. No pus followed; with a syringe, a warm boracic acid solution was injected into the sinus and found ready egress through the ostimen maxillary into the lett naris, but bringing with it no pus. This was rather disconcerting, but before abandoning our diagnosis a trochar was forced with slight resistence into the antrum through the pocket of the posterior root and upon withdrawal considerable pus followed. The cavity was then washed out with boracic acid solution but it was found that only with considerable difficulty could the solution be forced from this portion of the antrum into the naris. The reason was obvious; a bony septum divided the antrum into two chambers arranged anteroposteriorly. Natural communication existed but it was too slight to admit of good drainage. The diagnosis thus made clear, the alveolar opening was sufficiently enlarged by removal of the second bicuspid to enable us to break up this thin septum, good drainage was established and in due time the case was discharged cured ; although purulent discharge through the naris continued for several months after the operation and irrigation had to be practiced daily.

But while such cases offer additional difficulties it must not be forgotten that any chronic antral empyema is a most obstinate condition to cure. This is partly due to the difficulty of maintaining good drainage owing to a tendency on the part of the alveolar opening to close up, but chiefly, I think, to degenerative changes which occur in the lining epithelium after prolonged suppuration. Such a case I saw with Dr. House, seven or eight years ago, a mail carrier, in which not only both antra were effected but also the ethmoidal cells anterior and posterior on both sides. Everything possible was done to effect good drainage, but while the patient was much improved it is still necessary for him to syringe out the cavities once or twice daily to carry away the purulent secretion which is constant. The patients general health does not seem to suffer from this continued suppuration in most cases but I have no doubt that in the end such a condition must undermine resistence.

In regard to treatment of antral empyema little need be said at this time. The old rule which permits of no exception, requiring us to evacuate a collection of pus wherever and whenever found, holds good in this case. If we are certain that we have a collection of pus of any chronicity in the antrum the indications are plain it seems to me, seek drainage at the lowest possible point, make the opening sufficiently large for this purpose to permit also of exploration of the cavity.

To do this it is sometimes necessary to sacrifice one or two or even three sound teeth but it is better to do so if necessary than to be conservative at the expense of a prolonged suppurative disease with its consequent destruction of the delicate epithelial layer of the mucosa.

It sometimes happens that this operation is all sufficient to effect a cure but in my experience this is the exception. It more often becomes necessary to introduce a tube (made of gold or silver) attached to a vulcanized rubber plate to maintain the opening, through which the cavity may be frequently irrigated and medicated.

In perforating the bony plate between the alveolus and antrum a small trephine operated by a dental motor or foot engine I consider the best instrument, as giving the least pain (if an anesthetic is not used) and making the most favorable opening; a large dental drill or a strong trochar can also be successfully used for this purpose if the trephine is not obtainable.

Enlarging the ostium maxillare or opening the antrum in the inferior meatus are neither of them sufficient to insure the best results in chronic suppurative disease of this sinus, an alveolor opening of considerable size must be effected. For washing out the cavity, boracic acid solution (saturated) or normal saline solution are equally excellent and are to be preferred to the more powerful antiseptics. Under no circumstances is it justifiable to use peroxide of hydrogen for this purpose, for obvious, reasons, although formerly it was frequently employed. If suppuration is persistent the cavity may be loosely packed with iodoform gauze, or treated with other preparations of iodine; europhin and aristol are also recommended.

But after all, in these cases, the secret of success in treatment lies in securing and maintaining good drainage and cleanliness in keeping up the patients general health by careful attention to personal and dietary hygiene and guarding against complicating infections of the contiguous mucosa.

DISCUSSION.

Dr. Brophy: It has afforded me a great deal of pleasure to listen to a paper prepared and read by a general practitioner in medicine, dealing with the subject of empyema of the antrum in the able manner with which it has been done and which has taken into consideration the important part that pathology of the teeth bears to this disease.

Dr. King has presented a most excellent paper on this subject and he has convinced me that his knowledge is based not upon theory, but clinical experience which is always the most reliable knowledge that we have. The doctor has spoken of the pathology of the antrum, inception of disease, he has covered the phase of it which I think comes through intranasal infection and he has stated what I have always believed was the most common cause of antral abscess, or at least antral abscess most frequently arises from infection coming directly from diseased teeth. He has pointed out a phase in anatomy, an anomaly so to speak, yet it occurs quite frequently, perhaps it is not quite right to call it an anomaly, in the formation of bony divisions or septums dividing the antrum into different cavities. In this case under his direction he found in opening the anterior wall that no pus escaped, but going further back he found a quantity of pus near the distal wall. It so happens that the antrum is divided into various cavities something like a honeycomb, three, four or five different cavities, and as we extend towards the apex, in the malar process of the bone, we find numerous very small cavities reaching out into the malar process, and instead of one cavity normally we have from one to a dozen, any one of which may be the center of infection while the others may be free as in the case so well illustrated by Dr. King.

In the work of Professor Cryer, of Philadelphia, with which we are all familiar, he has pointed out that not infrequently the frontal sinus may discharge its contents directly into the antrum without first passing into the nasal cavity and the antrum in this manner be secondarily involved with infection. The ethymoidal cells may likewise discharge their contents directly into the antrum. In these cases the surgeon opens into antrum and takes out pus and treats the antrum antiseptically and by so doing believes he can effect a cure but the pus will continue to form. The first thing necessary is to get a large opening in order to make an ocular examination of the cavity. We must be able to see into the cavity. The X-ray may help us in the future but heretofore it has failed to reveal anything abnormal to us, so we must depend entirely upon an ocular examination. The opening should be made as large as the thumb. It is sometimes necessary to cut away the whole external wall of cavity so as to determine with what we are contending, we have all kinds of growth, and by so doing we may then determine whether the pus comes from the antral wall. If we can not arrest it with antiseptic treatments, and stimulate it with agents properly employed, we may at least discover that some accessory cavities are involved which will call for further surgical effort. After opening, then cleanse and remove all diseased bone and polypi or anything which would tend to keep up disturbance. It is especially gratifying to me to hear Dr. King tell us of the frequency of errors in diagnosis of gentlemen who give treatment for post nasal catarrh, believing the the patient was suffering from diseases of the nasal membranes when the disease was in the antrum. The excoriation of membranes in these cases was due to the passage of fluid from the antrum, this fluid is about as bad as dilute sulphuric acid in its irritating qualities.

It is not an easy matter to make a diagnosis of the antrum. There is a very simple way which I long ago detected to determine whether there is pus in the antrum, it is to keep patient upon affected side for several hours and then have them turn their head toward the healthy side, if the opening of the antrum into the nose is not closed the fluid will be quickly evacuated from the antrum and come out of the nose. Many of these patients have openings down through the distal part of the antrum into the nose and go on suffering through life without treatment, with supposed catarrh. I have an idea that thousands of people are suffering from antral abscess who are unconscious of the disease. Because it comes on slowly and goes on to the chronic form and unless it is very troublesome they tolerate it.

The removing of a tooth may be necessary, provided the tooth is bad and broken down, but if the tooth is nearly sound and could be restored to usefulness, I think it should be done. In order to get at the seat of the disease, lift up the cheek and with dental engine, which is better than the trocar because it is under perfect control, the operator knows what it will do, take a drill in the dental engine and carry through the canine fossa, if the fossa is present it will pass through very readily, if not it will do no particular harm providing the parts are aseptically clean. This may be done without any fear of further inconvenience to the patient. If pus be present it will quickly escape through the aperture and the surgeon may then go on and make opening into the affected parts.

A sarcomma may arise in back of the sphenoidal fissure and access to this tumor and its removal from within the mouth and antrum may be accomplished in almost all cases without external incision.

In almost all chronic cases, I do not now speak of those simple cases which may call for simple antiseptic treatment, but if we have polypi we should make a large opening and keep it so; there is one great difficulty we have to contend with, to retain this opening. To make a small opening and put in a little tube is a very unsatisfactory way in my estimation to treat chronic empyema of the antrum; because it means something more than the formation of pus upon the walls of the antrum. In almost all cases it means formation of polypi or disease of the associated sinuses and we cannot go along groping in the dark and throw fluids into the little opening with the hope of a patient getting better. I would advocate always then a liberal opening. Make it large enough to view all the parts. The incision should extend from the bicuspid back to the second molar, lifting up the muco-periosteum and cutting away the whole external wall thus giving a good view of the cavity. We can then remove the septums and put the parts in the proper condition. I was glad to hear the Doctor speak of pyrozones and hydrogen peroxid, these waters have done more harm to our patients than any agents ever produced, they are doing more harm than anybody will ever be able to tell because they do just as the Doctor stated. He cited a case where he had simple affection of the antrum and the effect it caused. These waters immediately upon coming into contact with the pus forms a gas and the affected material is carried into all the sinuses and establishes a secondary infection in remote places and harm is done. We have all had experiences where an alveolar abscess is formed and pyrozone is used, the gas is immediately formed and we have immediate pain, using these waters in confined areas is just like setting fire to powder. The pyrozone will expand and drive the infectious material into regions far distant. If carried down into a root it may pass out through the external alveolar plate, if not this it may pass underneath the periosteum and simply lift it away from bone and carry the infection along with it and not infrequently we find necrosis as a result. I have had a great deal of experience in surgical clinics and I know it will do this very thing. I think of all agents introduced into the dental profession there is nothing that has done as much harm as pyrozone and oxygenated waters. There are other agents which are far better. Silver nitrate is being used now in these cases. I have used protargal recently with quite a good deal of satisfaction. I used it in five and ten percent solutions. It is sometimes a difficult matter to maintain a large opening, but any dentist can make a canula of rubber so it will fit all the walls of the cavity. The introduction of gauze is all right after the first operation but later becomes objectional and it is then a source of infection.

Dr. Taft: Dr. Brophy has considered this subject in all its various phases and I hardly know what to say about it. There are one or two points that I would like to emphasize in connection with the importance of diagnosis, not as to locality merely but so far as general system is concerned. It is very certain that disease such as has been described in the paper and as referred to by Dr. Brophy, is much influenced in its progress and the characteristic stages which it passes through by the systemic condition of the patient, every one who has had experience in these cases will recognize this fact. Wherever there is a general cachexia it will produce its impressions upon the diseases of the antrum. Oftentimes the disease seems to increase even under proper treatment during unfavorable systemic conditions, so I have believed for a long time that it is a very important matter to determine and ascertain so far as possible the systemic condition. Both have sufficiently emphasized the necessity and importance of local diagnosis, in determining the condition in other features of these cases, in order that we may act intelligently in regard to the treatment.

In any case of diseased antrum the operator should know certainly the phase of the disease with which he has to deal, whether it is a mere engorgement of purulent matter, pus formation, or new growths, etc.

These things should all be plainly understood before proceeding with any treatment. Understand as fully as possible the systemic diathesis of the patient, his susceptibilities, and these with a local examination of the conditions and proper diagnosis will put one in a position to treat the case intelligently and succesfully.

Dr. Parker: A case came under my observation a few years ago of a young lady for whom I was treating a second bicuspid with a dead pulp which I had taken out and was getting the tooth into a condition to fill. I could see no apparent trouble about it especially but there was a great deal of soreness and pain about the face resulting finally in a badly swollen face. I

extracted the tooth, but as soon as I saw the end of the root, I knew that it was not the cause of the trouble; but I could not locate it, although I knew it was something else. I told her to go home and come back again in few days. She went home and I did not hear anything from her for about two weeks and I then heard that she had been almost at death's door with a terribly swollen face. I knew at once that there was something there which needed the attention of a dentist rather than a doctor. When I next saw her she had two openings on the outside of the face. I said my dear girl you have some antrum trouble. On further examination I found an abscess on the molar tooth which had extended into the antrum. I extracted the tooth and made an opening up into the antrum and found a great deal of pus which on taking out brought immediate relief. I syringed the cavity out with dilute boracic acid and packed it with iodoform gauze and sent her away and told her to come back again in two days. She did so and I found a material change, the discharge through the face had ceased. I treated it about two weeks with a result that it healed up and to-day there is no special marking on the face.

This was a case in which I was at fault in my first diagnosis, if I had discovered the cause in that molar I could have saved her all that trouble and severe illness. I cite this case to impress upon the younger members of this society the care that they should exercise in diagnosis.

Dr. Brophy: I think Dr. King and myself have been misunderstood about the treatment of these cases. The drawing on the board would tend to mislead one, we do not mean by this drawing that the operator removes the section of the face in order to get into the antrum, but we lift up the cheek and get access above the teeth into the antrum, the operation is not outside but inside the mouth.

Dr. Oakman: I can not let this interesting subject pass without saying a word. I am pleased to have the Doctor state that the majority of the most purulent cases are caused by the teeth, I believe that to be so and have thought so for years, but I have had considerable opposition to contend with, many of our local physicians claiming it was due more often to nasal affections and catarrhal diseases. I noticed however that one of the recent editions of Appleton's year books contained statistics that the majority of purulent antrum cases were caused by the teeth. I have seen many cases which have been treated all the way from five to twelve years, one for fifteen years, for catarrh and they proved eventually to be antral troubles. This last case was a bad case of empyema of the antrum with extensive necrosis extending from the cuspid to the molar and in order to cure the case it was necessary to remove the necrosed bone.

I think quite frequently the frontal sinus is the primary cause and when this sinus becomes engorged with pus as Dr. Brophy has so plainly stated it may pass directly into the antrum. These cases are very close akin to brain surgery. I would rather Dr. Brophy should take all of these operations upon the frontal sinus. Where the case is purulent I think it requires more than a medicinal treatment. The whole inner membrane of the antrum should be curetted and as Dr. Brophy also stated the opening should be made large, large enough to explore into the antrum with the index finger. I believe it was a dentist, who recommended poulticing the face for an antral trouble;

I think that is the next thing to if not criminal practice. I remember very well a case at college of a boy six years of age where it was necessary to remove the entire lower jaw because of a physician having recommended poulticing the face for an abscess on the lower jaw.

I think that the cases where we find double antral trouble are very rare, they are usually unilateral. Dr. Taft has spoken of overestimation, I think this is possible as much so as underestimation, but I think where one is overestimated there are twenty which do not receive estimation enough. When they are overestimated I think it occurs at the first sitting by the operater being too ambitious.

Dr. Brophy spoke of gauze being used immediately after the operation, of course this is always necessary, but I stick to the further use of it, provided, of course, I can see my patient daily. The introduction of a rubber canula sufficiently large may be a good thing, but the pus will not only pass down through the canula into the mouth, but is also likely to leak down the outside of the canula and get into the mouth and be carried into the stomach, which may cause gastro-intestinal disturbances. This is especially true at night time. So I maintain, from my slight observation, that it is good practice, if we can see the patient daily to tampoon the antrum, leaving the packing in too long of course is dangerous. If the gauze is packed in the antrum it absorbs the pus and each day when you remove the gauze you remove the pus which would have passed into the stomach. I think this much the best thing and I can not help disagreeing with my honored professor. In most all branches of oral surgery I endeavor to follow in his footsteps but this is one case where I can not. I have had no experience in the use of protargal, there is nothing much better than boracic acid. Glyco thymoline is also an excellent thing to use.

Dr. King closes the discussion: There is very little to be said in conclusion, since Dr. Brophy and others have covered most of the points in their admirable discussions. I might refer again to the importance of looking up the constitutional habits of the individual before diagnosing any case. This is quite true in the case which I cited as the subsequent history showed. This young man had a discharge from the antrum for several months he was cured after the operation so far as the antrum was concerned but in six month after that he developed tuberculosis of the tonsils which is not a common manifestation of tuberculosis. He was under my care from that time until his death which occurred some weeks later from absorption of infected material from the tonsils and he died of tubercular meningitis. It has always been my opinion that his original antral trouble was tubercular.

It is not the normal condition of the mucous membrane to suppurate and when it continues to do so after primary cause has been removed you may be sure that the case is constitutional. If you have suppuration in the region of a molar tooth and after removing it the case continues to suppurate, then you are forced to the conclusion that there is something in the antrum which is instrumental in keeping up that suppuration. On examination it will be found to be polypus or some unhealthy granulation of mucous membrane or some other condition calling for further surgical interference. These are always surgical and not medicinal.

There is a certain amount of danger in giving too much treatment along conservative lines but I do not think this is a common fault, the other extreme is far more likely.

